# Clinical characteristics and outcome of COVID-19 patients with Parkinson’s disease: a hospital-based case–control study in Shanghai, China

**DOI:** 10.3389/fnagi.2023.1138418

**Published:** 2023-05-05

**Authors:** Li Wu, Jun Shen, Yuhan Jiang, Xiaolei Shen, Ping Wang, Xin Nie, Wenyan Kang, Jianren Liu, Wei Chen

**Affiliations:** ^1^Department of Neurology, Shanghai Ninth People’s Hospital, Shanghai Jiao Tong University School of Medicine, Shanghai, China; ^2^Biostatistics Office of Clinical Research Unit, Shanghai Ninth People’s Hospital, Shanghai Jiao Tong University School of Medicine, Shanghai, China; ^3^Department of Neurology, Ruijin Hospital, Shanghai Jiao Tong University School of Medicine, Shanghai, China; ^4^Ruijin-Hainan Hospital, Shanghai Jiao Tong University School of Medicine (Hainan Boao Research Hospital), Shanghai, Hainan, China

**Keywords:** COVID-19, Omicron, Parkinson’s disease, clinical characteristics, outcome

## Abstract

**Background:**

Clinical manifestations of Parkinson’s disease (PD) after Corona Virus Disease 2019 (COVID-19) infection are poorly investigated.

**Objective:**

We aimed to explore the clinical features and outcomes of hospitalized PD patients with COVID-19.

**Methods:**

A total of 48 PD patients and 96 age-and sex-matched non-PD patients were included. Demographics, clinical characteristics and outcomes were compared between two groups.

**Results:**

PD patients with COVID-19 were elderly (76.69 ± 9.21 years) with advanced stage (H-Y stage 3–5 as 65.3%). They had less clinical symptoms (nasal obstruction, etc.), more proportions of severe/critical COVID-19 clinical classification (22.9 vs. 1.0%, *p* < 0.001), receiving oxygen (29.2 vs. 11.5%, *p* = 0.011), antibiotics (39.6 vs. 21.9%, *p* = 0.031) therapies, as well as longer hospitalization duration (11.39 vs. 8.32, *p* = 0.001) and higher mortality (8.3% vs. 1.0%, *p* = 0.001) relative to those without PD. Laboratory results showed that the PD group had higher white blood cell counts (6.29 vs. 5.16*10^9^, *p* = 0.001), neutrophil-to-lymphocyte ratio (3.14 vs. 2.11, *p* < 0.001) and C-reactive protein level (12.34 vs. 3.19, *p* < 0.001).

**Conclusion:**

PD patients with COVID-19 have insidious clinical manifestation, elevated proinflammatory markers and are prone to the development of severe/critical condition, contributing to a relatively poor prognosis. Early identification and active treatment of COVID-19 are pivotal to advanced PD patients during the pandemic.

## Introduction

Coronavirus disease 2019 (COVID-19), caused by the novel severe acute respiratory syndrome coronavirus 2 (SARS-CoV-2), has spread globally and become a pandemic for more than 2 years since December 2019 (Huang et al., 2020). After the virus mutated several times, the new Omicron variant of SARS-CoV-2 was identified in South Africa since November, 2021 (Viana et al., 2022). In February 2022, an Omicron outbreak happened in Shanghai, and the city had to be locked down due to the increasing number of infections. There were approximately 626,863 Omicron infectors during the pandemic (Zhang et al., 2022).

Certain pre-existing diseases and aging appeared to be associated with more severe COVID-19 manifestations, raising the attention toward Parkinson’s disease (PD), a common central neurodegenerative disease (Williamson et al., 2020; Bouali-Benazzouz and Benazzouz, 2021; COVID-19 Forecasting Team, 2022). However, data about the impact of COVID-19 on PD patients showed conflicting results. Some papers have also shown that a SARS-CoV-2 infection may lead to idiopathic parkinsonism (Cohen et al., 2020; Faber et al., 2020; Méndez-Guerrero et al., 2020), while α-synuclein and antiparkinsonian drug amantadine may be protective against COVID-19 (Beatman et al., 2015; Ait Wahmane et al., 2020; Grieb et al., 2021). Overexpression of α-synuclein in PD patients has been shown to effectively block the neurotropic invasiveness of SARS-CoV-2 (Ait Wahmane et al., 2020). In addition, case reports have shown that PD patients taking amantadine do not exhibit typical clinical features of COVID-19 after infection with SARS-CoV-2 (Grieb et al., 2021). So far, there are very few studies focused on the clinical manifestations in confirmed COVID-19 patients who have had pre-existing diagnoses of PD.

Studies have shown that COVID-19 positive PD patients were prone to an exacerbation of motor symptoms, and they exhibited typical clinical features of COVID-19, such as fever, nasal congestion, loss of smell and so on (Fasano et al., 2020). Nevertheless, some studies have also shown that PD patients presented respiratory symptoms atypically when infected with SARS-CoV-2 (Hainque and Grabli, 2020). Furthermore, PD patients have been associated with high COVID-19-related mortality, ranging from 5.2 to 100%(Fearon and Fasano, 2021). The poor prognosis and increased mortality of PD during the pandemic need to be addressed urgently to reduce the burden on the majority of patients and their families. Unfortunately, the majority of the previous studies were based on community-based patients and telephone surveys (Cilia et al., 2020; Fasano et al., 2020; Santos-García et al., 2020; Fathi et al., 2022). Both suffer the risk of bias as more severe, and longer-duration patients may be missed through this approach.

Hence, the present report aimed to investigate the clinical characteristics, outcomes, and laboratory findings associated with hospitalized PD patients infected by the Omicron variant from a Chinese population in Shanghai, China.

## Methods

### Participants

This is a retrospective case–control study including 48 PD patients and 96 sex-and age-matched non-PD patients with COVID-19 who were admitted to a government designated hospital for COVID-19 treatment in Shanghai during the Omicron wave from April to June 2022. Clinical physical examination was performed and the diagnosis of PD were confirmed by two experienced neurologists who worked in the designated hospital, according to the latest Movement Disorder Society (MDS) diagnostic criteria for PD (Postuma et al., 2015). All participants were diagnosed with COVID-19 infection according to positive reverse-transcription polymerase chain reaction (RT-PCR) for SARS-CoV2. SARS-CoV-2 viral genomes’ phylogenetic characteristics showed that all of the new viral genomes in Shanghai were clustered into the SARS-CoV-2 BA.2.2 sublineage. The study was approved by the Medical Ethics Committee of Shanghai Ninth People’s Hospital and Shanghai Jiao Tong University School of Medicine, Shanghai, China.

### Data collection

A standardized questionnaire was used to obtain information for all the cases, including demographics (age and sex), duration of onset symptoms to hospitalization, clinical symptoms (typical symptoms: cough, expectoration, fever, sore throat, runny nose, and nasal obstruction; gastrointestinal symptoms: poor appetite, diarrhea, nausea, vomiting, and abdominal pain; nervous system symptoms: fatigue, myalgia, headache, dizziness, smell impairment, taste impairment, emotional disorder, vision impairment, impairment consciousness, etc.), vaccination status, comorbidities (hypertension, diabetes, cardiovascular disease, chronic renal disease, chronic liver disease, chronic lung disease, cognitive impairment, and mood disorders), ancillary tests, treatments and outcomes. Questionnaires were conducted by the doctors working at the designated hospital. Disease severity was classified as asymptomatic, mild, moderate, severe, or critical, based on the latest version of the national COVID-19 protocol (National Health Commission and the General Office of National Administration of Traditional Chinese Medicine, 2022).

Laboratory parameters (blood routine, liver and kidney function, myocardial enzymes and coagulation function), chest computed tomography (CT) results, treatment regimens, and prognosis (hospitalization duration, viral shedding time and death) were documented and extracted via the inpatient electronic medical history.

For the PD patients, we additionally recorded disease duration, type of oral anti-parkinsonian medications, levodopa equivalent daily dose (LEDD) (Tomlinson et al., 2010), and motor complications. We also assessed the disease severity with the modified Hoehn and Yahr (H&Y) stage and classified the patients into early (H&Y 1–2.5) and advanced (H&Y 3–5) PD, according to the national consensus (Chen et al., 2016).

### Statistical analysis

SPSS version 23.0 was used for the statistical analysis. Categorical variables were presented as frequencies/percentages and compared with the *x*^2^-test or Fisher’s exact test. Continuous variables were presented as “mean ± SD” or medians (interquartile ranges) and then compared using the independent *t*-test or the Mann–Whitney *U*-test. Significance was set at a *p* < 0.05.

## Results

### General characteristics of PD patients

As shown in Table 1, 27 PD patients (56.3%) were female, the mean age of patients was 76.96 ± 9.21 years, and the disease duration was 8.02 ± 5.80 years. The proportion of patients in the advanced group (H&Y stage range 3–5) was 64.6% and the LEDD was 643.82 ± 391.54 mg. Among those PD patients, 2 (4.2%) cases previously received deep brain stimulation (DBS), and moreover, a significant proportion of them developed motor complications: wearing-off in 5 (10.4%) patients and dyskinesia in 3 (6.3%) patients. 16 (33.3%) patients had dysphagia. Overall, the majority of COVID-19 patients with PD were elderly and advanced patients.

**Table 1:** General characteristics of COVID-19 patients with PD.

Items	COVID-19 with PD (*n* = 48)
Age (years)	76.96 ± 9.21
Sex	
Male	21 (43.8%)
Female	27 (56.2%)
Disease duration (years)	8.02 ± 5.80
Hoehn and Yahr stage	
1–2.5	17 (35.4%)
3–5	31 (64.6%)
Drug	
Levodopa	42 (87.5%)
Dopamine agonists	30 (62.5%)
COMT inhibitors	12 (25.0%)
MAO-B inhibitors	15 (31.3%)
Amantadine	12 (25.0%)
LEDD (mg)	634.82 ± 391.54
DBS	2 (4.2%)
Dysphagia	16 (33.3%)
Wearing-off	5 (10.4%)
Dyskinesia	3 (6.3%)

COVID-19, Corona Virus Disease 2019; PD, Parkinson’s disease; COMT, catechol-O-methyl transferase; MAO-B, monoamine oxidase B; LEDD, Levodopa equivalent daily dose; DBS, deep brain stimulation.

### Clinical characteristics of PD patients with COVID-19

Owing to the matching process, the age and gender distributions were not statistically different between the PD and non-PD groups (*p =* 0. 360 and *p =* 1.000, respectively). Their demographic and clinical characteristics are shown in Table 2.

**Table 2:** Clinical characteristics and treatment outcomes of COVID-19 patients with PD.

Items	COVID-19 without PD (*n* = 96)	COVID-19 with PD (*n* = 48)	*p*-value
Age, years	77.69 ± 10.81	76.96 ± 9.21	0.360
Sex			1.00
Male	42 (43.8)	21 (43.8)	
Female	54 (56.2)	27 (56.2)	
Comorbidities			
Any, *n* (%)	81 (84.4)	43 (89.6)	0.454
Hypertension, *n* (%)	54 (56.3)	19 (39.6)	0.055
Diabetes, *n* (%)	23 (24.0)	13 (27.1)	0.688
Cardiovascular disease, *n* (%)	37 (38.5)	17 (36.2)	0.855
Chronic renal disease, *n* (%)	8 (8.3)	3 (6.4)	0.752
Chronic liver disease, *n* (%)	3 (3.1)	0 (0)	0.551
Chronic lung disease, *n* (%)	22 (22.9)	4 (8.3)	0.038
Cognitive impairment, *n* (%)	13 (13.5)	5 (10.4)	0.790
Mood disorders, *n* (%)	5 (5.2)	10 (20.8)	0.007
No. of comorbidities			
0	16 (16.7)	4 (8.3)	0.277
1	25 (26.0)	17 (35.4)	–
≥2	55 (57.3)	27 (56.3)	
COVID-19 vaccination status			0.014^*^
Unvaccinated, *n* (%)	65 (67.7)	42 (87.5)	
Vaccinated, *n* (%)	31 (32.3)	6 (12.5)	
Duration of onset symptoms to hospital admission	3 (1,7)	3 (1,5)	0.923
Symptoms on admission			
Cough, *n* (%)	74 (77.1)	33 (68.8)	0.415
Expectoration, *n* (%)	56 (58.3)	24 (51.1)	0.474
Fever, *n* (%)	40 (41.7)	16 (34.0)	0.466
Sore throat, *n* (%)	31 (32.3)	14 (29.2)	0.849
Runny nose, *n* (%)	26 (27.1)	8 (16.7)	0.213
Nasal obstruction, *n* (%)	17 (17.7)	2 (4.2)	0.034^*^
Anorexia, *n* (%)	18 (18.8)	3 (6.3)	0.049^*^
Diarrhea, *n* (%)	13 (13.5)	3 (6.3)	0.264
Nausea, *n* (%)	8 (8.3)	0 (0)	0.052
Vomiting, *n* (%)	6 (6.3)	0 (0)	0.179
Abdominal pain, *n* (%)	2 (2.1)	1 (2.1)	1.000
Fatigue, *n* (%)	20 (20.4)	6 (12.5)	0.259
Myalgia, *n* (%)	22 (22.9)	4 (8.3)	0.038^*^
Dizziness, *n* (%)	17 (17.7)	1 (2.1)	0.007^**^
Headache, *n* (%)	12 (12.5)	2 (4.2)	0.143
Olfactory dysfunction, *n* (%)	7 (7.3)	1 (2.1)	0.269
Decreased consciousness, *n* (%)	2 (2.1)	4 (8.3)	0.095
Clinical classification			<0.001^***^
Asymptomatic, *n* (%)	4 (4.2)	0 (0)	
Mild, *n* (%)	71 (74.0)	24 (50.0)	
Moderate, *n* (%)	20 (20.8)	13 (27.1)	
Severe/critical, *n* (%)	1 (1.0)	11 (22.9)	
Treatment			
Oxygen therapy, *n* (%)	11 (11.5)	14 (29.2)	0.011^*^
Nasal cannula oxygen	10 (10.4)	9 (18.8)	0.194
Face mask oxygen	1 (1.0)	3 (6.3)	0.108
Invasive mechanical ventilation	1 (1.0)	3 (6.3)	0.108
Antibiotic, *n* (%)	21 (21.9)	19 (39.6)	0.031^*^
Glucocorticoids, *n* (%)	5 (5.2)	9 (18.8)	0.015^*^
Intravenous immunoglobulin, *n* (%)	1 (1.0)	4 (8.3)	0.042^*^
Anticoagulation, *n* (%)	20 (20.8)	14 (29.2)	0.301
Thymosin, *n* (%)	15 (15.6)	10 (20.8)	0.487
Nutritional support, *n* (%)	28 (29.2)	18 (37.5)	0.346
Antiviral (paxlovid), *n* (%)			0.625
None	51 (53.1)	27 (56.3)	
Half amount	8 (8.3)	2 (4.2)	
Full amount	37 (38.5)	19 (39.6)	
Clinical outcome			
Hospitalization duration	8.32 ± 3.12	11.39 ± 6.97	<0.001^***^
Turning to nucleic acid negative duration	10 (8,13)	10 (6,14)	0.563
Mortality, *n* (%)	1 (1.0)	4 (8.3)	0.001^**^

^*^p < 0.05; ^**^p < 0.01; ^***^p < 0.001.

In terms of pre-existing diseases, compared with those without PD, COVID-19 patients with PD were more likely to be associated with mood disorders (20.8% vs. 5.2%, *p =* 0.007) and were less likely to be combined with chronic lung disease (8.3% vs. 22.9%, *p =* 0.038). However, there were no differences in the case of other pre-existing diseases, including hypertension, diabetes, cardiovascular disease, chronic renal disease, chronic liver disease and cognitive impairment. The majority of patients with PD had not been vaccinated (*n =* 42, 87.5%), while the vaccinated were more common in the non-PD group relative to the PD group (32.3% vs. 12.5%, *p =* 0.014).

Concerning clinical symptoms, PD patients were less likely to have anorexia (6.3% vs. 18.8%, *p* = 0.049), nasal obstruction (4.2% vs. 17.7%, *p* = 0.034), myalgia (8.3% vs. 22.9%, *p* = 0.038) and dizziness (2.1% vs. 17.7%, *p* = 0.007) symptoms. No significant differences were observed in symptoms such as cough, fever, sore throat, runny nose, headache, olfactory dysfunction or decreased consciousness. Therefore, when infected with the Omicron variant, the clinical manifestations of PD patients were relatively insidious.

**Figure 1:**
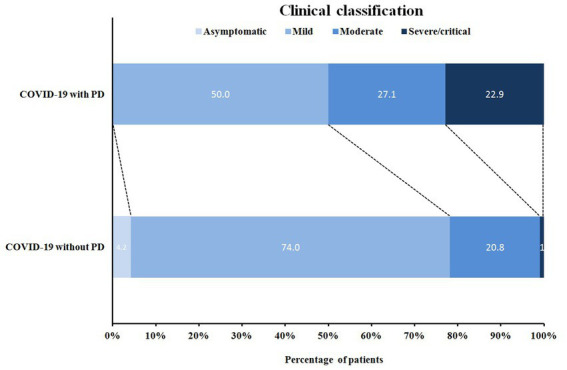
Distribution of clinical classification in COVID-19 patients with/without PD. COVID-19 with PD: COVID-19 patients with Parkinson’s disease (*n* = 48); COVID-19 without PD: COVID-19 patients without Parkinson’s disease (*n* = 96).

**Figure 2:**
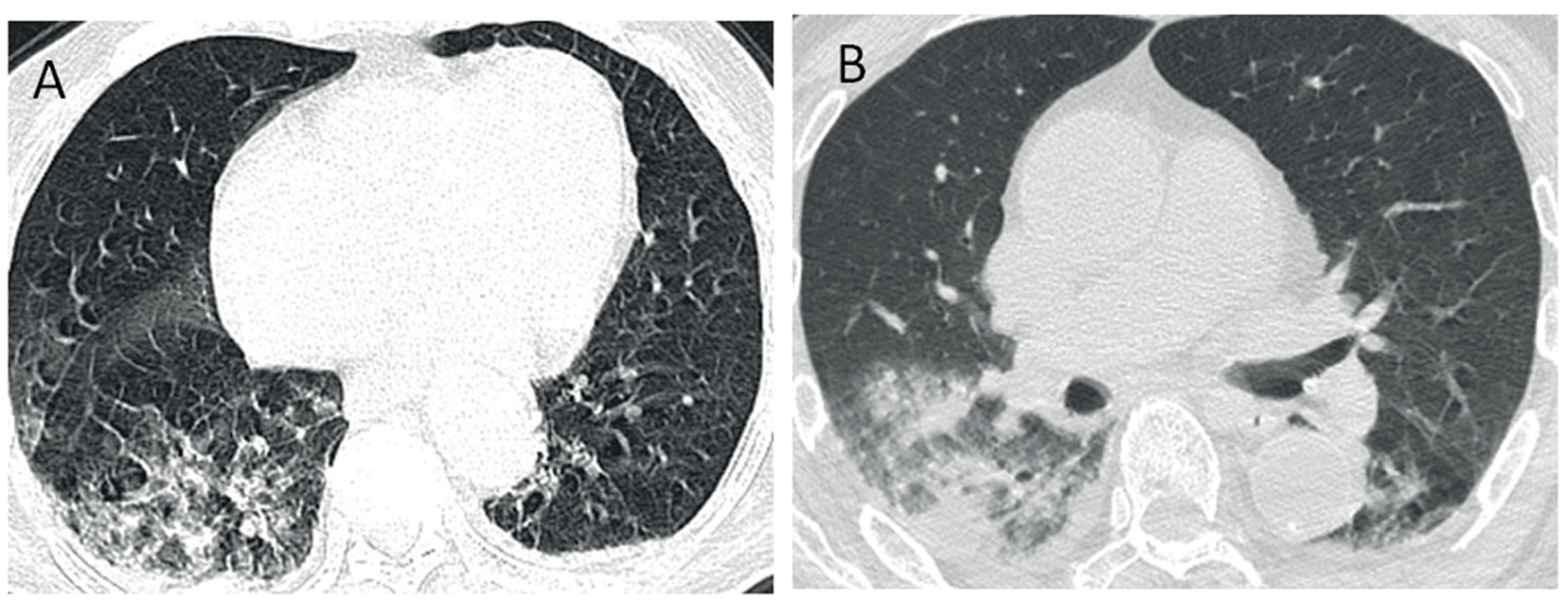
Chest CT images of a COVID-19 patient with PD. Axis chest CT scan showed bilateral multiple ground-glass opacities **(A)** on admission and consolidation of the right lung and pleural effusion **(B)** progressed within 48 h, consistent with typical COVID-19 infection. PD, Parkinson’s disease.

According to the latest national guidelines for COVID-19, the clinical classification of COVID-19 patients with PD was more severe when compared to non-PD (*p* < 0.001) (Figure 1). Inevitably, the severe/critical type of COVID-19 showed ground-glass opacity, effusion shadowing and even large-area consolidation, as evident from chest CT images of a representative PD patient, as shown in Figure 2.

Regarding clinical treatment, the PD group received more oxygen therapy (29.2% vs. 11.5%, *p* = 0.011), more use of antibiotics (39.6% vs. 21.9%, *p* = 0.031), glucocorticoids (18.8% vs. 5.2%, *p* = 0.015) and immunoglobulins (8.3% vs. 1.0%, *p* = 0.042).

In terms of clinical outcomes, the hospitalization duration was longer (11.39 ± 6.97 vs. 8.33 ± 3.12 *p* < 0.001) and the mortality rate was higher (8.3% vs. 1.0%, *p* < 0.001) for the PD group. However, the median time from onset of symptoms to hospital admission was 3 days and there was no statistical difference between the two groups. Patients in the PD group had poor outcomes, which was not associated with waiting time for hospitalization.

### Laboratory findings in PD patients with COVID-19

Table 3 contains information about the laboratory results, which showed that the PD group had a higher white blood cell count (6.29 vs. 5.16 × 10^9^, *p* = 0.001), higher neutrophil count (4.32 vs. 2.79 × 10^9^, *p* = 0.001), lower lymphocyte count (1.33 vs. 1.38 × 10^9^, *p* = 0.010), higher proportion of neutrophils (67.33% vs. 57.61%, *p* = 0.049), lower proportion of lymphocytes (21.53% vs. 28.20%, *p* = 0.036), a higher neutrophil-to-lymphocyte ratio (3.14 vs. 2.11, *p* < 0.001) and higher C-reactive protein levels (12.34 vs. 3.19, *p* < 0.001).

**Table 3:** Laboratory findings of COVID-19 patients with PD.

Laboratory finding	COVID-19 without PD (*n* = 96)	COVID-19 with PD (*n* = 48)	*p*-value
White blood cell count, *10^9^/L	5.16 ± 1.79	6.29 ± 2.87	0.001^**^
Neutrophil count, *10^9^/L	2.79 (2.07, 3.82)	4.32 (2.47, 7.08)	0.001^**^
Lymphocyte count, *10^9^/L	1.38 ± 0.52	1.33 ± 0.95	0.010^*^
Neutrophil, %	57.61 ± 12.45	67.33 ± 16.52	0.049^*^
Lymphocyte, %	28.20 ± 10.48	21.53 ± 14.30	0.036^*^
Monocyte count, *10^9^/L	0.55 ± 0.18	0.48 ± 0.21	0.601
Hemoglobin, g/L	129.72 ± 19.45	123.69 ± 16.51	0.307
Platelet count, *10^9^/L	194.49 ± 81.11	187.72 ± 71.02	0.952
Neutrophil/Lymphocyte	2.11 (1.40, 3.08)	3.14 (1.87, 10.73)	<0.001^***^
Monocyte/Lymphocyte	134.72 (102.13,200.58)	166.67 (103.00,256.36)	0.665
Creatinine, μmol/L	73.50 (61.00, 88.75)	79.00 (69.00, 101.00)	0.113
BUN, mmol/L	5.10 (4.30, 6.38)	6.14 (4.90, 7.70)	0.015^*^
Albumin, g/L	39.47 ± 4.24	37.25 ± 6.31	0.063
AST, μ/L	24.50 (19.25, 31.75)	25 (17.25, 39.00)	0.459
ALT, μ/L	16.00 (11.25, 30.75)	16.00 (10.00, 24.00)	0.458
LDH, U/L	207.00 (178.00, 233.00)	225.50 (185.00, 282.50)	0.202
Creatine Kinase, U/L	85.00 (57.50, 127.50)	88.50 (62.00, 132.75)	0.473
Myohemoglobin, ng/mL	28.00 (19.65, 49.05)	49.35 (32.28, 73.65)	0.002^**^
Troponine T, ng/mL	0.010 (0.004, 0.015)	0.018 (0.006, 0.027)	0.041^*^
Troponine I, ng/mL	0.006 (0.004, 0.012)	0.008 (0.004, 0.017)	0.116
PT, s	11.04 ± 0.87	11.07 ± 0.68	0.186
APTT, s	28.04 ± 2.99	29.73 ± 4.59	0.053
Fibrinogen, g/L	3.19 ± 0.93	3.45 ± 1.11	0.704
D-dimer, mg/L	0.46 (0.23, 0.85)	0.56 (0.27, 1.26)	0.245
C-reactive protein, mg/L	3.19 (1.47, 7.03)	12.34 (1.82, 40.31)	<0.001^***^
Lactic acid, mmol/L	1.31 (1.05, 1.71)	1.20 (0.90, 1.80)	0.631
Erythrocyte sedimentation rate, mm/h	17.50 (8.25, 28.50)	21.00 (5.25, 40.75)	0.745

^*^p < 0.05; ^**^p < 0.01; ^***^p < 0.001.

## Discussion

As far as our knowledge is concerned, this is the first report in China on epidemiological data of hospitalized PD patients with Omicron variant infection. Our hospital-based case–control study revealed that (1) most of the PD patients with Omicron infection were elderly and mainly in the advanced stage; (2) PD patients with COVID-19 had insidious clinical manifestations, but relatively poor prognosis with a higher likelihood of transformation into severe/critical condition, required more oxygen and drugs therapy and suffered relatively higher mortality (8.3%); and (3) serum inflammatory markers were observed to increase in the abovementioned group of patients.

Our results demonstrated that PD patients infected with SARS-CoV-2 were less likely to develop typical COVID-19 symptoms, and some were even statistically different (nasal obstruction, anorexia, myalgia and dizziness). As of now, very few studies have demonstrated the clinical manifestation in PD patients with/without COVID-19. Fasano et al. observed that the clinical manifestation of COVID-19 largely overlapped with that of non-PD patients (Fasano et al., 2020). A clinical report of PD patients treated with DBS presented atypical symptoms of COVID infection (Hainque and Grabli, 2020). The above findings were very consistent with our study. Analyzing the reasons suggested that symptoms of COVID-19 such as fatigue, hyposmia, myalgia, and dizziness were also identified as PD symptoms. What we recorded were the new symptoms after the diagnosis of COVID-19 against the backdrop of the underlying disease. Therefore, in the context of COVID-19, any deterioration in these senses would be subjective and may not be reliable. In addition, some patients quickly developed adverse consequences, such as severe disease or death, after the COVID-19 infection, as was expected in our study and a previous study (Hainque and Grabli, 2020), leading to a challenge of early symptom detection. Furthermore, it has been postulated that α-synuclein (a key protein in PD pathogenesis) and amantadine (a common PD drug) may protect against a SARS-CoV-2 infection (Beatman et al., 2015; Ait Wahmane et al., 2020; Grieb et al., 2021). A small part of the COVID-19-confirmed PD patients taking amantadine did not show any typical symptoms (Rejdak and Grieb, 2020; Aranda-Abreu et al., 2021). All these factors could explain the insidious clinical manifestation in the cohort.

Our study could precisely confirm that PD patients infected with SARS-CoV2 were more likely to present severe/critical types of COVID-19, resulting in a relatively poor outcome. These findings were consistent with the reports from other countries. Artusi et al. (2020) *observed* that six out of eight hospitalized COVID-19 confirmed PD patients died in Italy. Antonini et al. showed that when infected with SARS-CoV-2, 4 out of 10 PD patients developed severe respiratory problems and subsequently died from COVID-19 related pneumonia in the United Kingdom (Aranda-Abreu et al., 2021). Mortality data for COVID-19 associated with PD patients was inconclusive, with values ranging from 5.2 to 100% (Fearon and Fasano, 2021). It is well known that certain pre-existing conditions and aging appear to be associated with more severe clinical symptoms. Thus, elderly, advanced PD patients form a subgroup of particularly vulnerable individuals (Williamson et al., 2020; COVID-19 Forecasting Team, 2022). Additionally, stiffness of respiratory muscles, impaired cough reflex, and pre-existing dyspnea resulted in increased severity of COVID-19 in patients with PD (Antonini et al., 2020). Currently, Omicron has replaced Delta as the predominant strain of COVID-19 in circulation. A cohort study revealed that the risk of serious outcomes following Omicron infection was much lower than that of Delta in England (Nyberg et al., 2022). As is evident, the overall mortality rate of Omicron infection is relatively low at present, but the case of PD combined with Omicron infection has not been reported so far in any report. Our article is the first in this regard. Unfortunately, the prognosis of PD patients infected with Omicron was quite unsatisfactory. Further analysis suggested that patients who died were older and the disease duration was longer, even though the comparison did not reach a statistical difference (Table 4). In addition, cases of death were reported to have more PD complications, especially wearing-off. Difficulty in swallowing also meant that patients were more likely to develop lung infections, leading to severe pneumonia. As shown in Table 1, the incidence of dysphagia in PD patients was 33.3%, which inevitably led to a higher occurrence of pneumonia in those individuals.

**Table 4:** Demographic and clinical features of the PD patients according to mortality.

Items	Dead *n*= 4)	Survival *n*= 44)	*p*-value
Age (years)	82.00 ± 6.63	76.50 ± 9.33	0.159
Sex			1.000
Male	2 (40%)	19 (43.2%)	
Female	3 (60%)	25 (56.8%)	
Age			0.338
<76 years	1 (25%)	24 (54.5%)	
≥76 years	3 (75%)	20 (45.5%)	
Disease duration (years)	12.00 ± 5.42	7.66 ± 5.75	0.846
Hoehn and Yahr stage			0.282
1–2.5	0	17 (38.6%)	
3–5	4 (100%)	27 (61.4%)	
Motor Complications			
Wearing-off	2 (50%)	3 (6.8%)	0.049*
Dyskinesia	1 (25%)	2 (4.5%)	0.234
LEDD (mg)	1046.25 ± 307.01	597.41 ± 379.23	0.278
COVID-19 Clinical classification			<0.001**
Mild	0	24 (54.5%)	
Moderate	0	13 (29.5%)	
Severe/Critical	4 (100%)	7 (15.9%)	
COVID-19 vaccination status			0.526
Unvaccinated	4 (100%)	32 (84.2%)	
Vaccinated	0 (0%)	6 (15.8%)	

^*^p < 0.05; ^**^p < 0.001.

Laboratory results suggested that when infected with COVID-19, the PD patients experienced a heightened inflammatory response, including higher white blood cell count and neutrophil count, lower lymphocyte count, a higher neutrophil/lymphocyte ratio (N/L) and CRP levels (Table 3). Mao and colleagues also found more inflammatory marker changes in patients with severe infection compared to those with a non-severe infection (Mao et al., 2020). Studies have shown that N/L is an early warning indicator for severe illness, and similarly, LDH is an early warning indicator for critical illness (Liang et al., 2020). Our results also discovered a trend toward higher LDH levels in the PD group, although not statistically significant. In addition, the levels of urea nitrogen and troponin T were also found significantly increased in the PD group when compared with the control. These results remind us of the fact that abnormal indicators of early warnings warrant more attention, thereby prompting a more rational use of medical resources with the aim to prevent the development of critical illnesses among hospitalized COVID-19 patients.

Studies have shown that vaccination against COVID-19 can significantly reduce the risk of severe illness and death. While analyzing the cause of death, it was found that all the deaths reported in this article were unvaccinated cases. Although the government’s propaganda is strong, PD patients have low vaccination rates. A recent study showed that the COVID-19 vaccination rate of PD was 54%, which was lower than that of the general elderly (Zhou et al., 2022). Impressively, the results reported that the COVID-19 vaccination acceptance rate of PD in Shanghai was much lower as compared to the nationwide (38.4% vs. 67.3%), due to its broader socioeconomic status, involving income, education and international exchanges (Zhou et al., 2022). Low COVID-19 vaccination rates were associated with multiple factors such as influenza vaccination history, duration of PD disease, geography and trust in vaccine effectiveness (Zhou et al., 2022). Integrating the above evidence, we recommend that PD patients should be vaccinated unless there were specific contraindications.

The limitations of the present study include small sample size and potential selection bias as all participants were from a single center. To investigate the natural course of PD patients after Omicron infection, a multi-center registry study during the pandemic in Shanghai is needed in future. In addition, we only evaluated the PD patients with H&Y stage. Detailed clinical features of PD, such as MDS-UPDRS and NMSQuest, were not assessed by the physicians. It will be of great value to explore the contributions of motor and non-motor symptoms to the clinical characteristics and treatment outcomes after COVID-19 infection in PD patients.

## Conclusion

The majority of COVID-19 patients with PD were elderly, advanced cases. PD patients with COVID-19 had insidious clinical manifestation, elevated serum inflammatory markers, and were vulnerable to the transformation into severe/critical conditions, contributing to a relatively poor prognosis. Early detection and active treatment of COVID-19 were found to be pivotal in treating advanced PD during the pandemic.

## Data availability statement

The original contributions presented in the study are included in the article/supplementary material, further inquiries can be directed to the corresponding authors.

## Ethics statement

The studies involving human participants were reviewed and approved by the Medical Ethics Committee of Shanghai Ninth People’s Hospital, Shanghai Jiao Tong University School of Medicine, Shanghai, China (2022-T130-2). The patients/participants provided their written informed consent to participate in this study.

## Author contributions

LW, JS, WK, WC, and JL designed the study. LW and WC handled the data analysis. JS, LW, PW, XS, YJ, XN, WK, WC, and JL participated in the collection and interpretation of data. LW and JS wrote the manuscript with input from all authors. All authors discussed the results and contributed to the final manuscript, revised the manuscript content, and approved the final version of the manuscript.

## Funding

This research was supported by grants from 200 talent project from Shanghai Municipal Education Commission-Gaofeng Clinical Medicine Grant Support (No. 20161422 to JL), Natural Science Foundation Project from the Shanghai Municipal Science and Technology Commission (No. 22ZR1436900 to JL), Clinical Research Program of Ninth People’s Hospital Affiliated to Shanghai Jiao Tong University School of Medicine (JYLJ202003 to WC), Project of Biobank from Shanghai Ninth People’s Hospital, Shanghai Jiao Tong University School of Medicine (YBKB202120 to WC), and Health Management Project of Shanghai Rehabilitation Medical Association (2022KJCX008 to WC).

## Conflict of interest

The authors declare that the research was conducted in the absence of any commercial or financial relationships that could be construed as a potential conflict of interest.

## Publisher’s note

All claims expressed in this article are solely those of the authors and do not necessarily represent those of their affiliated organizations, or those of the publisher, the editors and the reviewers. Any product that may be evaluated in this article, or claim that may be made by its manufacturer, is not guaranteed or endorsed by the publisher.

## References

[ref1] Ait Wahmane S. Achbani A. Ouhaz Z. Elatiqi M. Belmouden A. Nejmeddine M. (2020). The possible protective role of α-Synuclein against severe acute respiratory syndrome coronavirus 2 infections in patients with Parkinson’s disease. Mov. Disord. 35, 1293–1294. doi: 10.1002/mds.28185, PMID: 32519352 PMC7300655

[ref2] Antonini A. Leta V. Teo J. Chaudhuri K. R. (2020). Outcome of Parkinson’s disease patients affected by COVID-19. Mov. Disord. 35, 905–908. doi: 10.1002/mds.28104, PMID: 32347572 PMC7267273

[ref3] Aranda-Abreu G. E. Aranda-Martínez J. D. Araújo R. (2021). Use of amantadine in a patient with SARS-CoV-2. J. Med. Virol. 93, 110–111. doi: 10.1002/jmv.26179, PMID: 32542661 PMC7323182

[ref4] Artusi C. A. Romagnolo A. Imbalzano G. Marchet A. Zibetti M. Rizzone M. G.. (2020). COVID-19 in Parkinson’s disease: report on prevalence and outcome. Parkinsonism Relat. Disord. 80, 7–9. doi: 10.1016/j.parkreldis.2020.09.008 32920322 PMC7474816

[ref5] Beatman E. L. Massey A. Shives K. D. Burrack K. S. Chamanian M. Morrison T. E.. (2015). Alpha-synuclein expression restricts RNA viral infections in the brain. J. Virol. 90, 2767–2782. doi: 10.1128/JVI.02949-15, PMID: 26719256 PMC4810656

[ref6] Bouali-Benazzouz R. Benazzouz A. (2021). Covid-19 infection and parkinsonism: is there a link? Mov. Disord. 36, 1737–1743. doi: 10.1002/mds.28680, PMID: 34080714 PMC8242862

[ref7] Chen S. Chan P. Sun S. Chen H. Zhang B. Le W.. (2016). The recommendations of Chinese Parkinson’s disease and movement disorder society consensus on therapeutic management of Parkinson’s disease. Transl. Neurodegener. 5:12. doi: 10.1186/s40035-016-0059-z 27366321 PMC4928283

[ref8] Cilia R. Bonvegna S. Straccia G. Andreasi N. G. Elia A. E. Romito L. M.. (2020). Effects of COVID-19 on Parkinson’s disease clinical features: a community-based case-control study. Mov. Disord. 35, 1287–1292. doi: 10.1002/mds.28170, PMID: 32449528 PMC7280741

[ref9] Cohen M. E. Eichel R. Steiner-Birmanns B. Janah A. Ioshpa M. Bar-Shalom R.. (2020). A case of probable Parkinson’s disease after SARS-CoV-2 infection. Lancet Neurol. 19, 804–805. doi: 10.1016/S1474-4422(20)30305-7, PMID: 32949534 PMC7494295

[ref10] COVID-19 Forecasting Team (2022). Variation in the COVID-19 infection-fatality ratio by age, time, and geography during the pre-vaccine era: a systematic analysis. Lancet 399, 1469–1488. doi: 10.1016/S0140-6736(21)02867-1 35219376 PMC8871594

[ref11] Faber I. Brandão P. R. P. Menegatti F. Bispo D. D. C. Maluf F. B. Cardoso R. (2020). Coronavirus disease 2019 and parkinsonism: a non-post-encephalitic case. Mov. Disord. 35, 1721–1722. doi: 10.1002/mds.28277, PMID: 32815213 PMC7461093

[ref12] Fasano A. Cereda E. Barichella M. Cassani E. Ferri V. Zecchinelli A. L.. (2020). COVID-19 in Parkinson’s disease patients living in Lombardy. Italy. Mov Disord 35, 1089–1093. doi: 10.1002/mds.28176, PMID: 32484584 PMC7300944

[ref13] Fathi M. Taghizadeh F. Mojtahedi H. Jame S. Z. B. Moghaddam N. M. (2022). The effects of Alzheimer’s and Parkinson’s disease on 28-day mortality of COVID-19. Rev. Neurol. 178, 129–136. doi: 10.1016/j.neurol.2021.08.002, PMID: 34556345 PMC8435376

[ref14] Fearon C. Fasano A. (2021). Parkinson’s disease and the COVID-19 pandemic. J. Parkinsons Dis. 11, 431–444. doi: 10.3233/JPD-202320, PMID: 33492244 PMC8150477

[ref15] Grieb P. Świątkiewicz M. Prus K. Rejdak K. (2021). Amantadine for COVID-19. J. Clin. Pharmacol. 61, 412–413. doi: 10.1002/jcph.1802 33350472

[ref16] Hainque E. Grabli D. (2020). Rapid worsening in Parkinson’s disease may hide COVID-19 infection. Parkinsonism Relat. Disord. 75, 126–127. doi: 10.1016/j.parkreldis.2020.05.008, PMID: 32414669 PMC7205634

[ref17] Huang C. Wang Y. Li X. Ren L. Zhao J. Hu Y.. (2020). Clinical features of patients infected with 2019 novel coronavirus in Wuhan, China. Lancet 395, 497–506. doi: 10.1016/S0140-6736(20)30183-5, PMID: 31986264 PMC7159299

[ref18] Liang W. Liang H. Ou L. Chen B. Chen A. Li C.. (2020). China medical treatment expert group for COVID-19. Development and validation of a clinical risk score to predict the occurrence of critical illness in hospitalized patients with COVID-19. JAMA Intern. Med. 180, 1081–1089. doi: 10.1001/jamainternmed.2020.2033 32396163 PMC7218676

[ref19] Mao L. Jin H. Wang M. Hu Y. Chen S. He Q.. (2020). Neurologic manifestations of hospitalized patients with coronavirus disease 2019 in Wuhan, China. JAMA Neurol 77, 683–690. doi: 10.1001/jamaneurol.2020.1127, PMID: 32275288 PMC7149362

[ref20] Méndez-Guerrero A. Laespada-García M. I. Gómez-Grande A. Ruiz-Ortiz M. Blanco-Palmero V. A. Azcarate-Diaz R. J.. (2020). Acute hypokinetic-rigid syndrome following SARS-CoV-2 infection. Neurology 95, e2109–e2118. doi: 10.1212/WNL.0000000000010282, PMID: 32641525

[ref21] National Health Commission and the General Office of National Administration of Traditional Chinese Medicine (2022). Diagnosis and treatment protocol for COVID-9 patients (tentative 9th version). Infect. Dis. Immun. 2, 135–144. doi: 10.1097/ID9.0000000000000059 PMC9295936 37520110

[ref22] Nyberg T. Ferguson N. M. Nash S. G. Webster H. H. Flaxman S. Andrew N.. (2022). Comparative analysis of the risks of hospitalisation and death associated with SARS-CoV-2 omicron (B.1.1.529) and delta (B.1.617.2) variants in England: a cohort study. Lancet 399, 1303–1312. doi: 10.1016/S0140-6736(22)00462-7, PMID: 35305296 PMC8926413

[ref23] Postuma R. B. Berg D. Stern M. Poewe W. Olanow C. W. Oertel W.. (2015). MDS clinical diagnostic criteria for Parkinson’s disease. Mov. Disord. 30, 1591–1601. doi: 10.1002/mds.26424 26474316

[ref24] Rejdak K. Grieb P. (2020). Adamantanes might be protective from COVID-19 in patients with neurological diseases: multiple sclerosis, parkinsonism and cognitive impairment. Mult. Scler. Relat. Disord. 42:102163. doi: 10.1016/j.msard.2020.102163, PMID: 32388458 PMC7190496

[ref25] Santos-García D. Oreiro M. Pérez P. Fanjul G. Gonzálezet J. M. P. Painceiras M. J. F.. (2020). Impact of coronavirus disease 2019 pandemic on Parkinson’s disease: a cross-sectional survey of 568 Spanish patients. Mov. Disord. 35, 1712–1716. doi: 10.1002/mds.28261, PMID: 32776601 PMC7436468

[ref26] Tomlinson C. L. Stowe R. Patel S. Rick C. Gray R. Clarke C. E. (2010). Systematic review of levodopa dose equivalency reporting in Parkinson’s disease. Mov. Disord. 25, 2649–2653. doi: 10.1002/mds.23429, PMID: 21069833

[ref27] Viana R. Moyo S. Amoako D. G. Tegally H. Scheepers C. Althaus C. L.. (2022). Rapid epidemic expansion of the SARS-CoV-2 omicron variant in southern Africa. Nature 603, 679–686. doi: 10.1038/s41586-022-04411-y, PMID: 35042229 PMC8942855

[ref28] Williamson E. J. Walker A. J. Bhaskaran K. Bacon S. Bates C. Morton C. E.. (2020). Factors associated with COVID-19-related death using OpenSAFELY. Nature 584, 430–436. doi: 10.1038/s41586-020-2521-4, PMID: 32640463 PMC7611074

[ref29] Zhang X. Zhang W. Chen S. (2022). Shanghai’s life-saving efforts against the current omicron wave of the COVID-19 pandemic. Lancet 399, 2011–2012. doi: 10.1016/S0140-6736(22)00838-8, PMID: 35533708 PMC9075855

[ref30] Zhou Y. Lin Z. Wan X. Liu J. Ding J. Zhang C.. (2022). COVID-19 vaccine acceptance and hesitancy in patients with Parkinson’s disease. Fron Public Health. 10:977940. doi: 10.3389/fpubh.2022.977940, PMID: 36304248 PMC9595444

